# Retracted: Application Value of Real-Time Ultrasonic Elastograph with Serum Human Epididymis Protein 4, Interleukin-33, and Carbohydrate Antigen 153 in Diagnosis of Early Cervical Cancer

**DOI:** 10.1155/2023/9896428

**Published:** 2023-01-22

**Authors:** Journal of Healthcare Engineering

*Journal of Healthcare Engineering* has retracted the article titled “Application Value of Real-Time Ultrasonic Elastograph with Serum Human Epididymis Protein 4, Interleukin-33, and Carbohydrate Antigen 153 in Diagnosis of Early Cervical Cancer” [1] due to concerns that the peer review process has been compromised.

Following an investigation conducted by the Hindawi Research Integrity team [2], significant concerns were identified with the peer reviewers assigned to this article; the investigation has concluded that the peer review process was compromised. We therefore can no longer trust the peer review process, and the article is being retracted with the agreement of the Chief Editor.
